# TAZ Represses the Neuronal Commitment of Neural Stem Cells

**DOI:** 10.3390/cells9102230

**Published:** 2020-10-02

**Authors:** Natalia Robledinos-Antón, Maribel Escoll, Kun-Liang Guan, Antonio Cuadrado

**Affiliations:** 1 Instituto de Investigaciones Biomédicas “Alberto Sols” UAM-CSIC, 28029 Madrid, Spain; nrobledonos@iib.uam.es (N.R.-A.); mescoll@iib.uam.es (M.E.); 2 Instituto de Investigación Sanitaria La Paz (IdiPaz), 28029 Madrid, Spain; 3 Department of Biochemistry, Faculty of Medicine, Autonomous University of Madrid, 28029 Madrid, Spain; 4 Centro de Investigación Biomédica en Red sobre Enfermedades Neurodegenerativas (CIBERNED) ISCIII, 28031 Madrid, Spain; 5 Department of Pharmacology and Moores Cancer Center, University of California San Diego, La Jolla, CA 92093, USA; kuguan@health.ucsd.edu

**Keywords:** neural stem cells, neurogenesis, ASCL, NEUROG2, NEUROD1, SOX2, neuronal differentiation, hippo

## Abstract

The mechanisms involved in regulation of quiescence, proliferation, and reprogramming of Neural Stem Progenitor Cells (NSPCs) of the mammalian brain are still poorly defined. Here, we studied the role of the transcriptional co-factor TAZ, regulated by the WNT and Hippo pathways, in the homeostasis of NSPCs. We found that, in the murine neurogenic niches of the striatal subventricular zone and the dentate gyrus granular zone, TAZ is highly expressed in NSPCs and declines with ageing. Moreover, TAZ expression is lost in immature neurons of both neurogenic regions. To characterize mechanistically the role of TAZ in neuronal differentiation, we used the midbrain-derived NSPC line ReNcell VM to replicate in a non-animal model the factors influencing NSPC differentiation to the neuronal lineage. TAZ knock-down and forced expression in NSPCs led to increased and reduced neuronal differentiation, respectively. TEADs-knockdown indicated that these TAZ co-partners are required for the suppression of NSPCs commitment to neuronal differentiation. Genetic manipulation of the TAZ/TEAD system showed its participation in transcriptional repression of SOX2 and the proneuronal genes ASCL1, NEUROG2, and NEUROD1, leading to impediment of neurogenesis. TAZ is usually considered a transcriptional co-activator promoting stem cell proliferation, but our study indicates an additional function as a repressor of neuronal differentiation.

## 1. Introduction

In mammals, a subpopulation of embryonic neural precursors persists into adulthood as neural stem progenitor cells (NSPCs) and localizes at neurogenic niches, such as the subventricular zone (SVZ) of the striatum and the subgranular zone (SGZ) of the hippocampus. Despite some controversy in human studies [1], the general view is that NSPCs provide a source of neurons that may be relevant for the maintenance of brain functions, including cognition [2,3] and motor functions [4,5]. A common hallmark of aging is a progressive reduction of adult neurogenesis, which is accelerated in age-dependent neurodegenerative diseases. Therefore, the regulatory networks that control the dynamics of NSPCs in the SVZ and the SGZ are a subject of high interest in order to understand their participation in brain pathophysiology. These networks include the WNT and the Hippo signaling pathways which converge at the regulation of neural stem cells fate [6]. Transcriptional co-activators TAZ (transcriptional coactivator with PDZ binding motif) and YAP (Yes Associated Protein 1) are fundamental elements of these pathways and promote stem cell reactivation from quiescence [7,8,9]. Considering that YAP and TAZ appear to have overlapping but also distinct roles, in this study, we focused on TAZ because, compared to YAP, fewer studies have directly addressed its function [10,11]. TAZ stimulates expression of genes involved in cell growth and self-renewal, and uncontrolled TAZ activation contributes to tumorigenesis [12,13]. Although the implication of TAZ in cell proliferation and migration has been well established in cancer [14,15], little is known about its role in the regulation of NSPCs commitment to neuronal differentiation.

TAZ is part of the WNT signaling pathway, which participates in NSPC regulation at the SVZ and SGZ during development, in the adult and aging brain [4,16,17,18]. Neurogenesis is attenuated by the downregulation of WNT signaling with aging and in age-related neurodegenerative diseases such Alzheimer’s or Parkinson’s diseases [2,3,5]. In the absence of WNT activity, the destruction complex consisting of β-catenin APC, Axin, and GSK-3 targets TAZ for β-TrCP mediated ubiquitination and proteasome degradation, thus maintaining low TAZ levels. On the contrary, when WNT signaling is active, the destruction complex is not assembled and TAZ escapes proteasome degradation [19,20]. Then, TAZ migrates to the nucleus, binds transcription factors TEADs (TEA Domain Transcription Factors) [12], and regulates expression of its target genes. Several WNT transcriptional responses and biological effects are mediated by TAZ [21].

TAZ is also regulated by the Hippo pathway, which is a conserved protein serine/threonine kinase cascade that leads to the phosphorylation and inhibition of TAZ [22]. The Hippo pathway participates in development, differentiation, organ size, and regeneration [22,23]. When the Hippo pathway is inactive, unphosphorylated TAZ escapes this negative control and together with TEADs regulate the expression of its target genes.

Multipotent NSPCs undergo asymmetric divisions generating neural progenitors and neurons [24]. This process involves a number of key factors, including SOX2 (Sex-determining region Y (SRY)-related HMG box 2) and several proneuronal factors [25,26]. SOX2 marks the regulatory regions of the proneuronal genes for epigenetic regulation, thereby enabling appropriate activation of the neuronal differentiation program upon exposure to a neurogenic stimulus [27,28]. In vertebrates, the proneuronal factors comprise ASCL1 (Achaete-Scute complex-like 1), NEUROG2 (Neurogenin-2) and NEUROD1 (Neurogenic differentiation 1), among others. Transient expression of these proneuronal factors in NSPCs drives neuronal differentiation [29]. For example, ASCL1 is necessary to induce cell cycle exit and neuronal specification [30]. Together with NEUROG2, ASCL1 regulates the progression of retinal neurogenesis [31] and promotes deep-layer neurogenesis in the murine neocortex [32]. NEUROG2 also regulates cell cycle exit of neuronal progenitors [33]. NEUROD1 has been implicated with terminal differentiation, neuronal maturation, and survival [34,35,36,37].

In this study, we report the role of TAZ in the neuronal specification of NSPCs. In addition to the well-established function of TAZ as a co-activator of gene expression in stem cell reactivation, proliferation, and oncogenic transformation, we report here that TAZ participates in the repression of proneuronal genes and prevents the progression of NSPCs towards neuronal differentiation.

## 2. Materials and Methods

### 2.1. Animals

Mice were cared for according to protocols approved by the Ethics Committee for Research of the Universidad Autónoma de Madrid and by the Community of Madrid (PROEX 105/18).

### 2.2. Cell Culture and Reagents

HEK293T were maintained in Dulbecco’s Modified Eagle Medium supplemented with 10% fetal bovine serum (Sigma-Aldrich, St. Louis, MO, USA), 4 mM L-Glutamine (Gibco) and 80 mg/mL gentamicin (Laboratorios Normon, Madrid, Spain), 100 U/mL penicillin/streptomycin (Life Technologies, Grand Island, NY, USA) and 1% amphotericin B solution (Lonza, Hopkinton, MA, USA) in 5% CO_2_ at 37 °C conditions. The immortalized human neural stem cell line derived from ventral mesencephalon of fetal brain (ReNcell VM) was purchased from EMD Millipore (Billerica, MA, USA). ReNcells VM were plated onto Corning Matrigel hESC-Qualified Matrix (Corning, Bedford, MA, USA)-coated T75 cell culture flasks (BD Biosciences, San Jose, CA, USA) and maintained in neurobasal medium (Gibco, Thermo Fisher Scientific, Grand Island, NY, USA) supplemented with 2% (v/v) B27 supplement (Gibco), 20 ng/mL recombinant human epidermal growth factor (Peprotech, NJ, USA), 20 ng/mL recombinant human basic fibroblast growth factor (Peprotech, NJ, USA), 100 U/mL penicillin/streptomycin (Life Technologies, Grand Island, NY, USA) and 1% amphotericin B solution (Lonza, Hopkinton) in 5% CO_2_ at 37 °C conditions.

### 2.3. Lentiviral and Retroviral Vector Production and Infection

Pseudotyped lentiviral vectors were produced in HEK293T cells transiently co-transfected with 10 µg of the corresponding lentiviral vector plasmid, 6 µg of the packaging plasmid pSPAX2 (Addgene Watertown, MA, USA) and 6 µg of the VSV-G envelope protein plasmid pMD2G (Addgene) using Lipofectamine Plus reagent according to the manufacturer’s instructions (Invitrogen, Madrid, Spain). Retrovirus supernatant was prepared by transfection of HEK293T cells with 5 µg of each plasmid using Lipofectamine Plus. Lentiviral vector shRNA control (shco), shTAZ, and several shTEAD2 were purchased from Sigma-Aldrich. Lentiviral vector shTEAD1/3/4 was a generous gift from Zengqiang Yuan (Chinese Academy of Sciences, Beijing, China). The retroviral vectors used were: pBabePuro (Addgene), pBabePuroTAZ-WT, pBabePuroTAZ^S51A^ (TEAD-binding-defective mutant), pBabePuroTAZ^4SA^ (active TAZ with four serine residues in the HxRxxS motif replaced with alanine: S66A, S89A, S117A, and S311A), and pBabePuroTAZ^4SA+S51A^ (generous gifts of Prof. Kun-Liang Guan, Department of Pharmacology and Moores Cancer Center at University of California San Diego). Cells were infected in the presence of 4 µg/mL polybrene (Sigma-Aldrich) and selected with 0.5 µg/mL puromycin (Sigma-Aldrich).

### 2.4. Immunofluorescence

Thirty µm-thick coronal murine brain sections were processed for immunofluorescence microscopy as previously described [38]. Antibodies are shown in Table A1 of the Appendix A. Images were obtained using a Leica TCS SP5 confocal microscope and cell counts were performed using FiJi Software (ImageJ). ReNcells VM were adhered on Corning Matrigel hESC-Qualified Matrix coated coverslips, and fixed with 4% paraformaldehyde. Immunofluorescence was performed as described in [38]. Briefly, cells were washed, blocked in PBS containing 0.5% Triton X-100% and 3% bovine serum albumin and incubated for 16 h at 4 °C with the relevant primary antibodies and for 2 h at room temperature with the appropriate secondary antibodies coupled to Alexa Fluor 488, 555/546, or 647 (1:500) (Life Technologies-Molecular Probes, Grand Island, NY, USA). Nuclei were counterstained with DAPI. Images were quantified using the Fiji Software (http://fiji.sc/Fiji).

### 2.5. Differentiation and Neuron Complexity

ReNcell VM were plated after 5 days of lentiviral/retroviral infection on Corning Matrigel hESC-Qualified Matrix coated coverslips and incubated for 30 days in differentiation medium (Neurobasal medium supplemented with 2% (v/v) B27 supplement and antibiotics). Immunostaining and quantification were performed as described in [38]. Primary antibodies are described in Table A1 of the Appendix A. To quantify neuronal complexity, Sholl analysis was performed using the Simple neurite tracer plugin; total axonal, dendrite, or neurite length were determined using NeuronJ (https://imagescience.org/meijering/software/neuronj/) and the image processing package Fiji.

### 2.6. Immunoblotting

Immunoblotting was performed as described in [39]. Briefly, cells were homogenized in lysis buffer (TRIS pH 7.6 50 mM, 400 mM NaCl, 1 mM EDTA, 1 mM EGTA and 1% SDS) and samples were heated at 95 °C for 15 min, sonicated and pre-cleared by centrifugation for 10 min at 10,000 g. Proteins were resolved in SDS-PAGE, transferred to Immobilon-P (Millipore) membranes and detected with primary antibodies (Table A1 of the Appendix A). Proper peroxidase-conjugated secondary antibodies were used for detection by enhanced chemiluminescence (GE Healthcare, Chicago, IL, USA).

### 2.7. Chromatin Immunoprecipitation (ChIP)

This protocol was performed as described in [39]. Briefly, ReNcells MV were transfected with plasmid CT or pBabePuroTAZ^4SA^ encoding an active TAZ mutant [15]. The qPCR was performed from immunoprecipitated DNA with antibodies against TAZ RNA Polimerase II (Pol II) and acetyl histone H3 (AcH3). Quantitative PCR reactions were done with the primers shown in Table A2 of the Appendix A. Samples from 3 independent immunoprecipitations were analyzed.

### 2.8. Analysis of mRNA Levels

Total RNA extraction, reverse transcription and quantitative polymerase chain reaction (qRT-PCR) were done as detailed in [40]. Primer sequences are shown in Table A3 of the Appendix A. Data analysis was based on the ΔΔCT method, with normalization of the raw data by the geometric mean of to the housekeeping genes *ACTB*, *GAPDH* and *TBP* (Applied Biosystems). All PCRs were performed from triplicate samples.

### 2.9. MTT Assays

Reduction of MTT (3-(4,5-Dimethyl-2-thiazolyl)-2,5-diphenyl-2H-tetrazolium1 bromide) to its formazan salt was used as an estimation cell proliferation (Cell Proliferation Kit; Sigma-Aldrich). Briefly, 4000 cells/well were seeded in 96 well plates. At the time of analysis, cells were incubated with 1 mg/mL MTT for 2.5 h. The reaction was stopped by incubation in 100 µL DMSO for 20 min. Absorbance at 570 nm was taken as an indirect estimation of the proliferation rate of viable cells.

### 2.10. Statistical Analysis

Data are presented as mean ± S.D. or S.E.M. Differences between groups were analyzed using GraphPad Prism 5 software by one-way ANOVA or the unpaired Student’s *t*-test as indicated in the legends to figures.

## 3. Results

### 3.1. TAZ Expression Is Lost during Neuronal Differentiation

**Figure 1:**
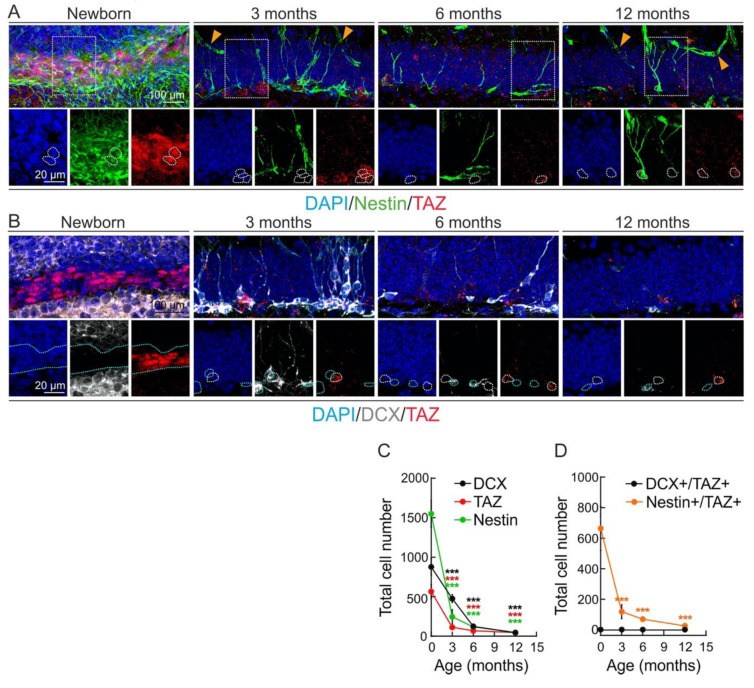
TAZ expression declines in the neurogenic niche of the subgranular zone (SGZ). (**A**,**B**), representative confocal immunofluorescence photographs of Nestin/TAZ and DCX/TAZ stained cells, respectively, in the SGZ of new-born, 3-, 6- and 12- month-old mice. Nuclei are counterstained with DAPI. White dotted lines indicate examples of TAZ^+^ cells. Yellow arrowheads denote Nestin-stained blood vessels that were not analyzed. Blue dotted lines indicate DCX^+^/TAZ^−^ cells. (**C**) quantification of Nestin^+^, DCX^+^ or TAZ^+^ cells. Data represent mean ± SEM (*n* = 5 mice per age). Asterisks denote statistically significant differences of the age 0 group vs. the other time points of DCX^+^ (black), TAZ^+^ (red), and Nestin^+^ (green) groups, according to one-way ANOVA. *** *p* < 0.001. (**D**) quantification of Nestin^+^/TAZ^+^ and DCX^+^/TAZ^+^ cells. Data represent mean ± SEM (*n* = 5 mice per age). Asterisks denote statistically significant differences of the age 0 group vs. the other time points of the Nestin^+^/TAZ^+^ groups, according to one-way ANOVA. *** *p* < 0.001. The changes in the DCX^+^/TAZ^+^ cells were not statistically significant.

**Figure 2:**
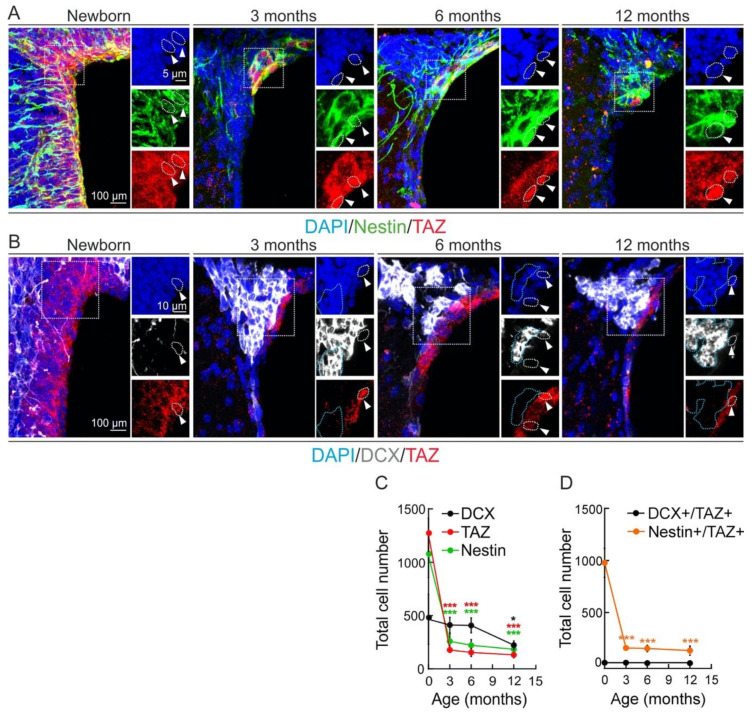
TAZ expression declines in the neurogenic niche of the subventricular zone (SVZ). (**A**,**B**), representative confocal immunofluorescence photographs of Nestin/TAZ and DCX/TAZ stained cells, respectively, in the SVZ of new-born, 3-, 6-, and 12- month-old mice. Nuclei are counterstained with DAPI. White arrowheads and dotted lines indicate TAZ^+^ cells. Blue dotted lines indicate DCX^+^/TAZ^−^ cells. (**C**), quantification of Nestin^+^, DCX^+^ or TAZ^+^ cells. Data represent mean ± SEM (*n* = 5 mice per age). Asterisks denote statistically significant differences of the age 0 group vs. the other time points of DCX^+^ (black), TAZ^+^ (red), and Nestin^+^ (green) groups, according to one-way ANOVA. * *p* < 0.05; *** *p* < 0.001 (**D**), quantification of Nestin^+^/TAZ^+^ and DCX^+^/TAZ^+^ cells. Data represent mean ± SEM (*n* = 5 mice per age). Asterisks denote statistically significant differences of the age 0 group vs. the other time points of the Nestin^+^/TAZ^+^ groups, according to one-way ANOVA. *** *p* < 0.001. The changes in the DCX^+^/TAZ^+^ cells were not statistically significant.

**Figure A1:**
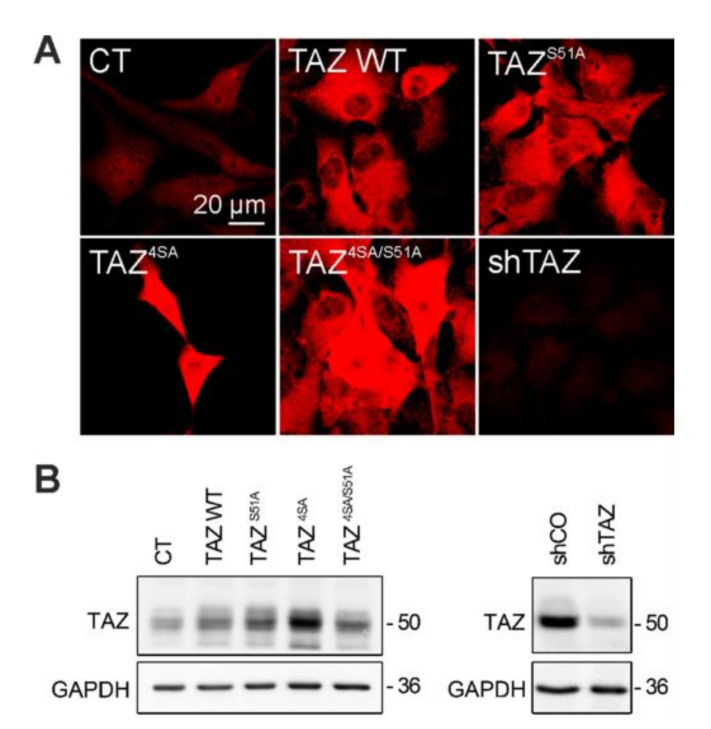
Specificity of the TAZ antibody. (**A**) Representative confocal images of TAZ immunostaining in cells transduced with empty viral vector (CT), or vector expressing wild type TAZ (TAZ-WT), or mutant TAZ^S51A^, TAZ^4SA^ and TAZ^4SA+S51A^ and shTAZ ReNcells VM after 5 days of viral transduction. (**B**) Representative immunoblot analysis of TAZ in ReNcells after 5 days of viral transduction, showing a specific band around 50 kDa. GADPH was used as load control.

We analyzed TAZ expression in the two main murine neurogenic niches, SGZ and SVZ, in new-born and 3-, 6-, and 12-month-old mice. We combined TAZ immunostaining with Nestin to identify NSPCs, or with doublecortin (DCX) to identify neuroblasts and immature neurons. The specificity of anti-TAZ antibody was validated in Figure A1 of the Appendix A. The pool of NSPCs (Nestin^+^ cells) and NSPCs-immature neurons (DCX^+^ cells) declined with aging in both the SGZ (Figure 1A–C) and the SVZ (Figure 2A–C), the decline in the SGZ being more evident and thus indicating specific features or each neurogenic zone. Moreover, TAZ-expressing cells also declined parallel to the exhaustion of the pool of progenitors (Figure 1C and Figure 2C). TAZ was expressed in Nestin^+^ cells but not in DCX^+^ cells, both in the SGZ (Figure 1D) and the SVZ (Figure 2D). Altogether, these results indicate that TAZ expression in the murine neurogenic niches is present in NSPCs and is progressively lost during neuronal differentiation.

**Figure 3:**
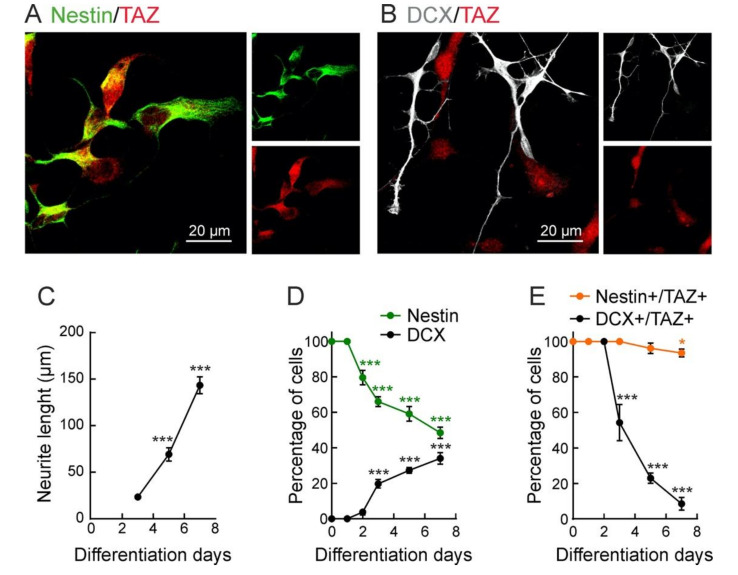
TAZ expression declines during neuronal differentiation. Representative confocal images of ReNcells VM immunostained with (**A**) Nestin and TAZ under proliferative conditions (in the presence of growth factors) or (**B**) immunostained with DCX and TAZ after seven days under differentiation conditions (in the absence of growth factors); (**C**) neurite length of DCX^+^ cells during differentiation; (**D**) quantification of Nestin^+^ and DCX^+^ ReNcells VM during differentiation; (**E**) quantification of Nestin^+^/TAZ^+^ cells DCX^+^/TAZ^+^ cells during differentiation. Data are mean ± S.E.M. (*n* = 50). Asterisks denote statistically significant differences of the group at age 0 vs. the other color-coded groups, according to one-way ANOVA. * *p* < 0.05, *** *p* < 0.001.

Considering that the dynamics of the NSPCs are most likely influenced by local niche factors, and the outcome on stemness, proliferation, and differentiation, is region-, age-, and cell-specific, in order to analyze the mechanistic regulation of NSPCs by TAZ in a general context, we used the midbrain-derived immortalized NSPC line ReNcell VM. These cells are an excellent tool to replicate, in a non-animal model, and, under controlled non-autonomous signals, the evolution of neurogenesis [41,42,43,44]. Under stem growth conditions (in the presence of growth factors), these cells expressed TAZ and also the NSPCs marker, Nestin, similar to the NSPCs of the neurogenic niches (Figure 3A). After 7 days in differentiation medium (in the absence of growth factors), many NSPCs were differentiated to immature neurons (DCX^+^) as determined by the progressive extension of neurites (Figure 3B,C). In parallel, we found a progressive reduction of Nestin^+^ NSPCs to ~50%, and a progressive increase of DCX^+^ in immature neurons to ~40% (Figure 3D). The loss of TAZ^+^ cells was further correlated with neuronal differentiation because the fraction of Nestin^+^/TAZ^+^ cells remained constant while that of DCX^+^/TAZ^+^ cells declined (Figure 3E). These results demonstrate a negative correlation between TAZ expression and exit of stemness towards neuronal differentiation, both in the mouse neurogenic niches and in the non-animal model of NSPCs.

### 3.2. TAZ Overexpression Represses Neuronal Differentiation of NSPCs

**Figure 4:**
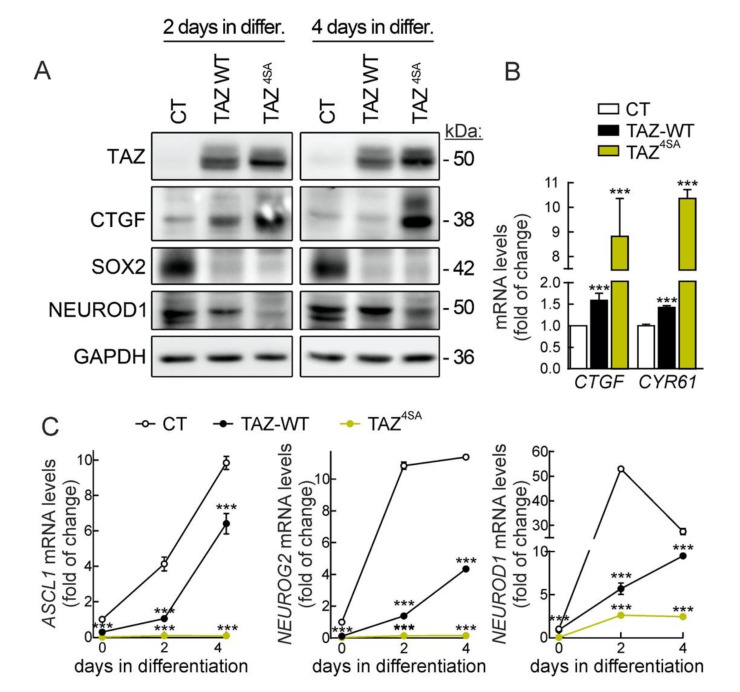
TAZ overexpression decreases the expression of SOX2 and proneuronal genes. (**A**) representative immunoblot of TAZ, CTGF, SOX2, NEUROD1, and GAPDH as a loading control in ReNcells VM transduced with wild type or TAZ^4SA^-expressing retrovirus after two or four days post infection; (**B**) mRNA levels of *TAZ* targets *CTGF* and *CYR61*; (**C**) mRNA levels of *ASCL1*, *NEUROG2* and *NEUROD1*. mRNA levels were determined by qRT-PCR and normalized by the geometric mean of *ACTB*, *GAPDH* and *TBP* levels. Data represent mean ± S.D. (*n* = 4). Statistical analysis was performed using one-way ANOVA. *** *p* < 0.001 vs. CT.

**Figure A2:**
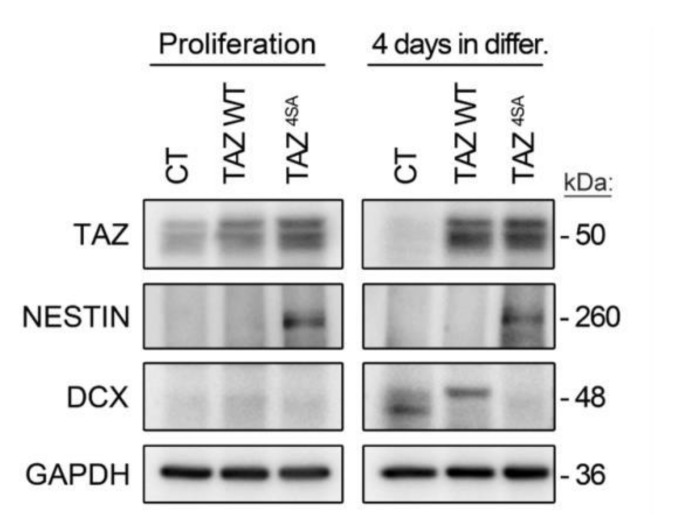
Effect of differentiation markers in ReNcells ectopically expression wild type or mutant TAZ4SA. ReNcells were transduced with retroviral expression vectors for wild type TAZ or mutant TAZ4SA under proliferating conditions (left panels). Then, cells were plated for 4 days in differentiation medium (right panels). Overexpression of the very active TAZ4SA mutant led to significant detection of NESTIN under proliferation conditions, but also in differentiation medium. By contrast, TAZ4SA blocked DCX expression under differentiator conditions.

We ectopically expressed wild type TAZ (TAZ-WT) or a very stable TAZ^4SA^ mutant harboring four Ser-to-Ala substitutions (S66A, S89A, S117A, S311A) that confer this protein constitutive activity because the LATS-induced inhibitory phosphorylation, leading to cytoplasmic retention and degradation, are abolished [22]. As shown in Figure A2 of the Appendix A, under proliferative conditions, but also under differentiation conditions, the expression of TAZ^4SA^ correlated with the expression of NESTIN, suggesting a block in exit from neural stemness. On the other hand, DCX was not detected under proliferative conditions, consistent with stemness, but TAZ^4SA^ also prevented DCX expression under differentiation conditions, indicating a block in neuronal differentiation. Moreover, under just two and four days in differentiation conditions, we found the expected accumulation of TAZ and its *bona fide* target CTGF (Figure 4A), and also *CTGF* and *CYR61* transcripts (Figure 4B). At the same time points, the protein levels of SOX2 and the proneuronal differentiation marker NEUROD1 declined (Figure 4A). The expression of other proneuronal factors, i.e., *ASCL1*, *NEUROG2,* or *NEUROD1* increased during differentiation in the control un-transduced cells (CT), while it remained low in TAZ-WT cells and were almost suppressed in TAZ^4SA^ cells (Figure 4C). These results suggest that either TAZ retains NSPCs in the stemness state or that it is a negative regulator of neurogenesis or both.

### 3.3. TAZ Depletion Favors Neuronal Differentiation of NSPCs

**Figure 5:**
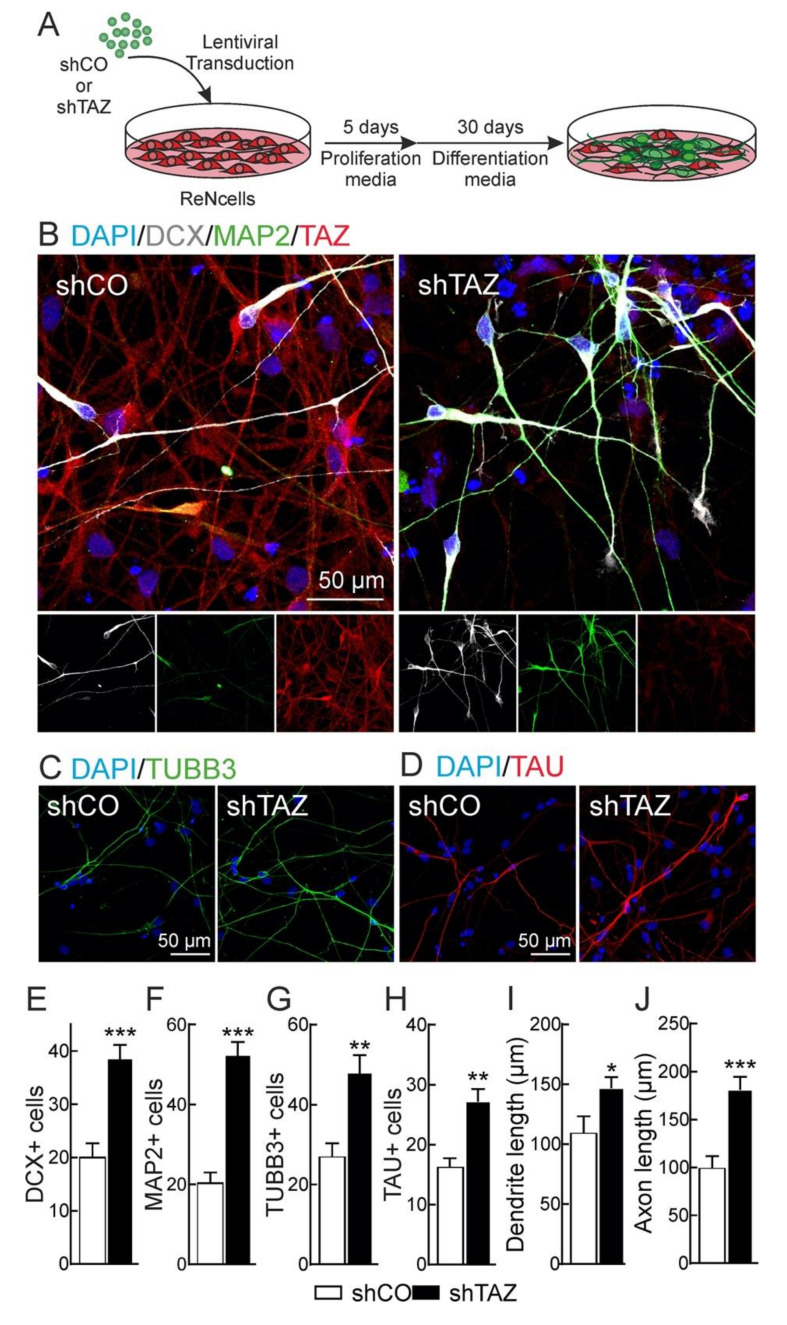
Loss of TAZ favours neuronal differentiation. (**A**) schematic overview of the experimental procedure. ReNcells VM were transduced with lentivirus encoding shcontrol (shCO) or human shTAZ, and after five days were plated under differentiation conditions (in the absence of growth factors) for 30 days. Representative confocal immunofluorescence photographs of DCX, MAP2, and TAZ (**B**), TUBB3 (**C**), and TAU (**D**). Nuclei are counterstained with DAPI. Quantifications correspond to DCX^+^ (**E**), MAP2^+^ (**F**), TUBB3^+^ (**G**), and TAU^+^ (**H**) cells. (**I**) Dendrite length based on MAP2 staining; (**J**) axonal length based on TAU staining. Data are mean ± S.E.M (*n* = 20). Statistical analysis was performed with the Student’s *t*-test. * *p* < 0.05; ** *p* < 0.01, *** *p* < 0.005.

**Figure A3:**
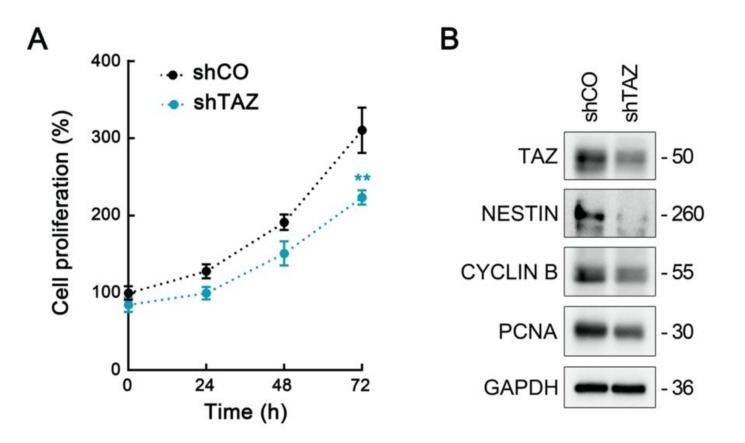
Analysis of proliferative markers in TAZ-depleted ReNcells. Cells were transduced with lentiviral vectors for expression of sh control (shCO) or shTAZ. (**A**) After 4 days in selection medium 4,000 cells/well of each condition were analyzed in quadrupled according to the MTT method (see Material and Methods). (**B**) At time 0, cell lysates were also analyzed by immunoblot. Note the decrease in the proliferative markers CYCLINB and PCNA as well as in the neural stem cell marker NESTIN.

The role of TAZ in proliferation of ReNcells VM was analyzed following TAZ knockdown. As shown in Figure A3 of the Appendix A, the proliferative rate and the expression of the proliferative markers CYCLIN B and PCNA, and the neural stem marker NESTIN were significantly reduced in TAZ-depleted cells, suggesting that TAZ is required to sustain cell proliferation and stemness of NSPCs. In order to further analyze the role of TAZ in neuronal differentiation, ReNcells VM were infected with a control lentivirus (shCO) or with a lentivirus for human TAZ-knockdown (shTAZ) (Figure A3 of the Appendix A), allowed to grow for five days under proliferative conditions, and then grown in differentiation medium for 30 days (Figure 5A). Neuronal differentiation was analyzed with DCX (neuroblasts and immature neurons), MAP2 (dendrites), TUBB3 (neurons), and TAU (axons) (Figure 5B–D). TAZ silencing resulted in an increase in neuronal number as determined by the quantification of DCX^+^ (Figure 5B,E), MAP2^+^ (Figure 5B,F), TUBB3^+^ (Figure 5C,G), and TAU^+^ (Figure 5D,H) cells. We next evaluated neuronal complexity and maturation as determined by dendrite length (MAP2 staining) and axonal length (TAU staining) by Sholl analysis. Comparing cell cultures with similar density, TAZ-knocked-down cells (shTAZ) presented slightly longer dendrites (Figure 5I) and axons (Figure 5J) than shCO cells. These results suggest that TAZ represses neuronal differentiation.

### 3.4. Transcription Factors TEAD Participate in TAZ Repression of Neuronal Differentiation

**Figure 6:**
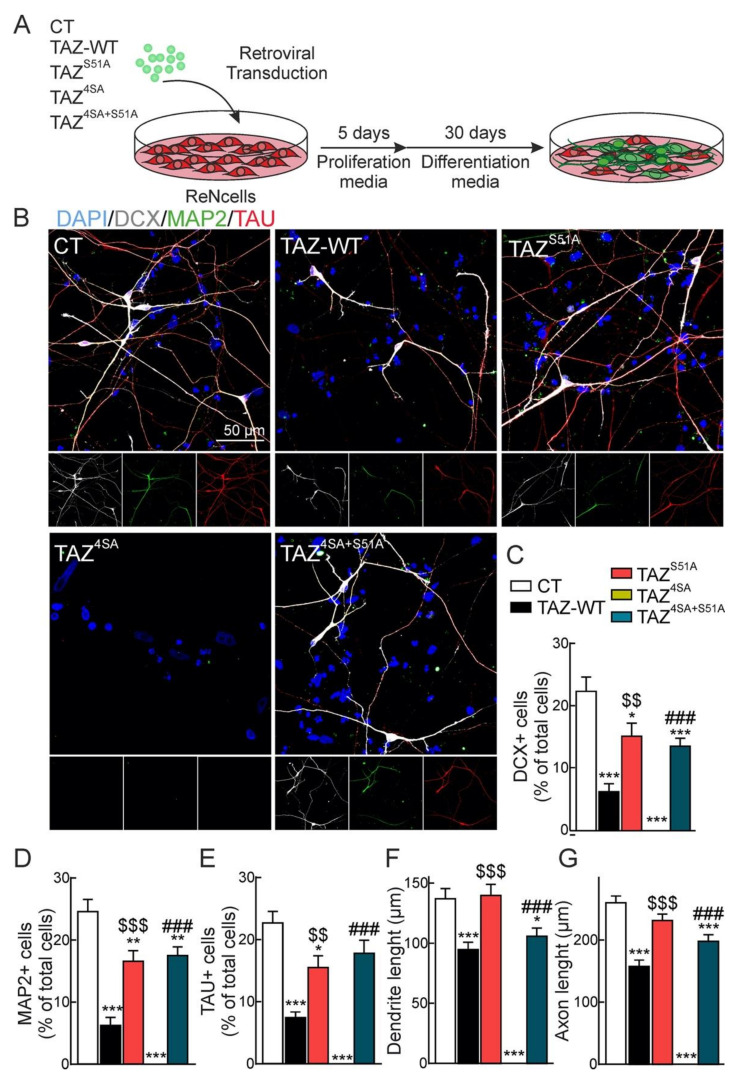
TEADs participate in the inhibitory effect of TAZ on neuronal differentiation. (**A**) schematic overview of the experimental design. ReNcells VM were transduced with empty vector (CT) or retroviral vector for overexpression of TAZ-WT and TAZ mutants TAZ^S51A^, TAZ^4SA^ and TAZ^4SA+S51A^. After five days of retroviral transduction, cells were incubated under differentiation conditions for 30 days; (**B**) immunostaining with neuronal markers DCX, MAP2, and TAU. Nuclei are counterstained with DAPI. Quantification of DCX^+^ (**C**), MAP2^+^ (**D**), and TAU^+^ (**E**) cells. (**F**) dendrite length based on MAP2 staining; (**G**) axonal length based on TAU staining. Data are mean ± S.E.M. (*n* = 20). Statistical analysis was performed with one-way ANOVA. * *p* < 0.05; ** *p* < 0.01, *** *p* < 0.001 vs. CT. $$ *p* < 0.01, $$$ *p* < 0.001 comparing TAZ^S51A^ vs. TAZ-WT. ### *p* < 0.001 comparing TAZ^4SA+S51A^ vs. TAZ^4SA^.

Transcriptional enhancer factor TEFs (TEADs) comprise a family of four paralogs that are the transcriptional co-partners of TAZ. Therefore, we tested the possible implication of TEADs in NSPCs fate as a counterpart for TAZ repression. We expressed wild type and several TAZ mutants [13]: single point mutant TAZ^S51A^ exhibits impaired binding to TEADs; TAZ^4SA^, described above, is constitutively active but retains binding to TEADs; TAZ^4SA+S51A^ has impaired TEADs binding [13] (Figure A1 of the Appendix A). ReNcells VM were transduced with retroviral vectors expressing these TAZ versions, maintained for five days in proliferation medium and then for 30 days in differentiation medium (Figure 6A). As shown in Figure 6B,C, overexpression of TAZ-WT led to a decrease in DCX^+^ cells, consistent with a role in repression of the neuronal programme. TAZ^4SA^ overexpression completely abolished neuronal differentiation and cells remained Nestin^+^ (data not shown). By contrast, TAZ^S51A^ had a modest effect, consistent with the need of TEAD co-partnering to repress neural differentiation. In line with this, TAZ^4SA+S51A^ expression did not block the neuronal differentiation. These observations were further confirmed with the analysis of two markers of neuronal differentiation, MAP2 and TAU (Figure 6D,E) and a Scholl analysis of neuronal complexity (Figure 6F,G). Overexpression of TAZ-WT, and more dramatically TAZ^4SA^, led to low levels of MAP2 and TAU as well as a reduced dendrite and axonal length compared to control cells. By contrast, overexpression of TEAD-binding defective mutants TAZ^S51A^ or TAZ^4SA+S51A^ had a very modest effect.

**Figure 7:**
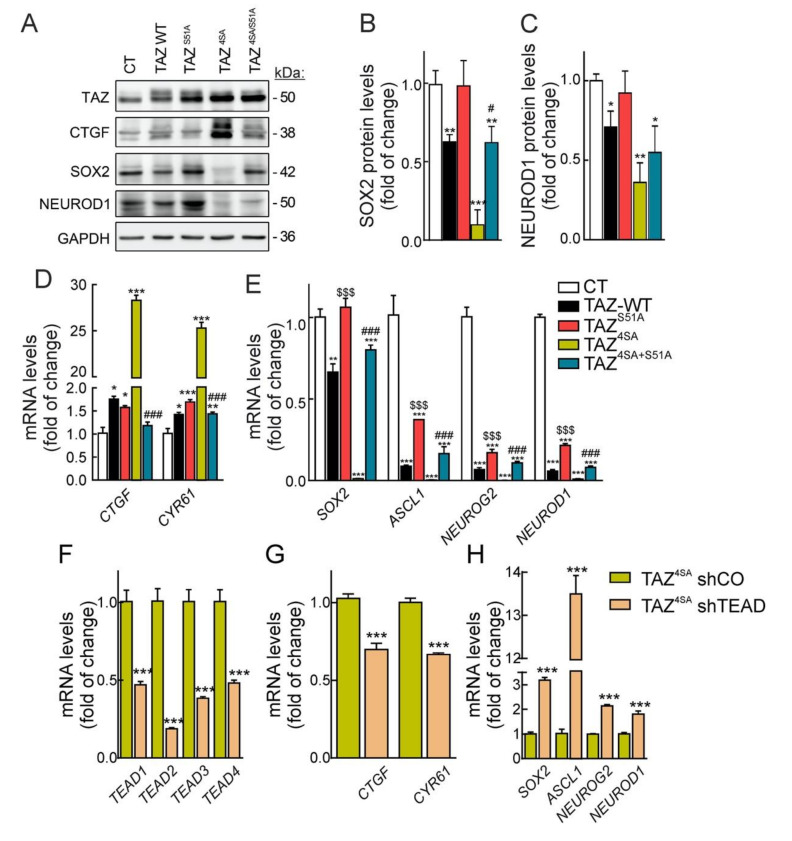
TAZ/TEAD is a transcriptional repressor of SOX2 and proneuronal transcription factors. (**A**) representative immunoblots of TAZ, CTGF, SOX2, NEUROD1, and GAPDH as a loading control in ReNcells VM CT, TAZ-WT, TAZ^S51A^, TAZ^4SA^, and TAZ^4SA+S51A^ after five days of retroviral transduction; (**B**) densitometric quantification of SOX2 protein levels in (**A**) relative to GAPDH; (**C**) densitometric quantification of NEUROD1 protein levels in (**A**) relative to GAPDH. Data are mean ± SEM (*n* = 4). (**D**) mRNA levels of TAZ targets *CTGF* and *CYR61*. (**E**) mRNA levels of *SOX2*, *ASCL1*, *NEUROG2* and *NEUROD1*. mRNA levels were determined by qRT-PCR and normalized by the geometric mean of *ACTB*, *GAPDH* and *TBP* levels. Data are presented as mean ± S.D. (*n* = 4). Statistical analysis was performed using one-way ANOVA. * *p* < 0.05; ** *p* < 0.01, *** *p* < 0.001 vs. control conditions. $$$ *p* < 0.001 comparing TAZ^S51A^ vs. TAZ-WT. # *p* < 0.05; ### *p* < 0.001 comparing TAZ^4SA+S51A^ vs. TAZ^4SA^. (**F**) mRNA levels of *TEAD1*, *TEAD2*, *TEAD3* and *TEAD4* in ReNcells VM after 5 days of lentiviral transduction with control vector (shCO) or shRNA against co-expression of shRNAs for TEAD1/3/4, and TEAD2 (shTEAD). (**G**) mRNA levels of *CTGF* and *CYR61*; (**H**) mRNA levels of *SOX2*, *ASCL1*, *NEUROG2,* and *NEUROD1*. mRNA levels were determined by qRT-PCR and normalized by the geometric mean of *ACTB*, *GAPDH* and *TBP* levels. Data represent mean ± S.D. (*n* = 4). Statistical analysis was performed using one-way ANOVA. *** *p* < 0.001 vs. shCO.

To gain more insight into the impact of the TAZ/TEAD partners in repression of neuronal differentiation, we further analyzed the expression of *SOX2* and the proneuronal genes *ASCL1*, *NEUROG2*, and *NEUROD1*. As expected, TAZ-WT and more intensely TAZ^4SA^ led to an increase in the TAZ target CTGF but also a decrease in SOX2 and NEUROD1 (Figure 7A–C). At the same time, these changes were not observed with the TAZ^S51A^ mutant, further pointing to the need of TEADs for the inhibition of proneuronal genes. At the level of transcription, TAZ-WT and more intensely TAZ^4SA^ increased the levels *CTGF* and *CYR61* (Figure 7D) and decreased the levels of *SOX2*, *ASCL1*, *NEUROG2*, and *NEUROD1*, this repression being almost complete in cells expressing TAZ^4SA^ (Figure 7E). By contrast, TAZ^S51A^ and TAZ^4SA+S51A^ did not affect *SOX2* expression and had a weak effect on *ASCL1*, *NEUROG2*, and *NEUROD1* compared to active TAZ^4SA^.

**Figure A4:**
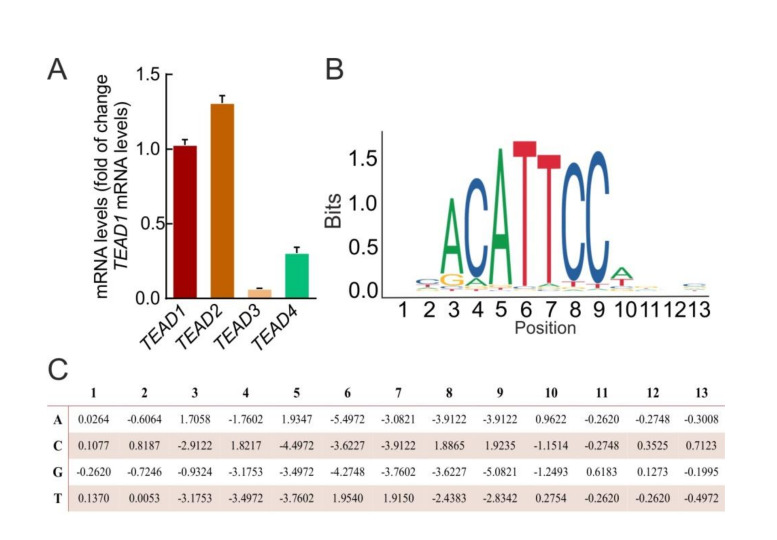
TEADs expression in ReNcells VM and JASPAR-based analysis of the consensus TEAD binding sequences. (**A**) mRNA levels of the *TEADs 1* to *4* were determined by qRT-PCR and normalized to the levels of *TEAD1*. mRNA levels were determined by qRT-PCR and normalized by the geometric mean of *ACTB*, *GAPDH* and *TBP* levels. Data represent mean ± S.D. (*n* = 4). (**B**) TEAD2 motif from JASPAR database indicating its consensus binding profile. (**C**) Position specific scoring matrix (PSSM) for TEAD2 PSSM derived from the frequency matrix available in JASPAR database (PSSM or TEAD1, TEAD3 and TEAD4 are available upon request).

We additionally knocked-down the TEAD isoforms in cells expressing TAZ^4SA^, i.e., with high TAZ repressor activity. As shown in Figure A4 of the Appendix A, NSPCs express mainly *TEAD1* and *TEAD2* which have been reported to cooperate in notochord maintenance as well as cell proliferation and survival in mouse development [45,46]. Knock-down of TEADs (Figure 7F) resulted in low levels of *CTGF* and *CYR61* transcripts as expected (Figure 7G), but, importantly, we observed the increase in *SOX2* and the *ASCL1*, *NEUROG2,* and *NEUROD1* proneuronal transcripts (Figure 7H), indicating that TAZ requires TEAD co-partners to exert repressor activity on neuronal differentiation.

### 3.5. Identification of Putative TAZ/TEAD-Interacting Regions in Proneurogenic Genes

We next determined if TAZ, through its transcription co-partners TEAD, might be directly involved in repression of SOX2 and the proneuronal factors ASCL1, NEUROG2, and NEUROD1. First, using the JASPAR database of consensus binding sequences for transcription factors [47] (Figure A4 of the Appendix A), we obtained the position specific scoring matrix (PSSM) for the sequences recognized by TEAD1, TEAD2, TEAD3, and TEAD4. Figure A4 of the Appendix A shows the scoring matrix for TEAD2 as an example. Then, we scanned *SOX2*, *ASCL1*, *NEUROG2,* and *NEUROD1* genes in search for putative TEAD-binding sites with a Python-based bioinformatics analysis (Appendix B). Table A4 of the Appendix A shows putative TEAD-binding sites in these genes assuming a relative score over 80%. According to the Encyclopedia of DNA Elements (ENCODE) of the human genome [48], many of these sites were located at DNase hypersensitive regions or in segments with acetylated histone H3 in Lysine 27 (H3K27Ac), both features being characteristic of open chromatin in regulatory regions.

**Figure 8:**
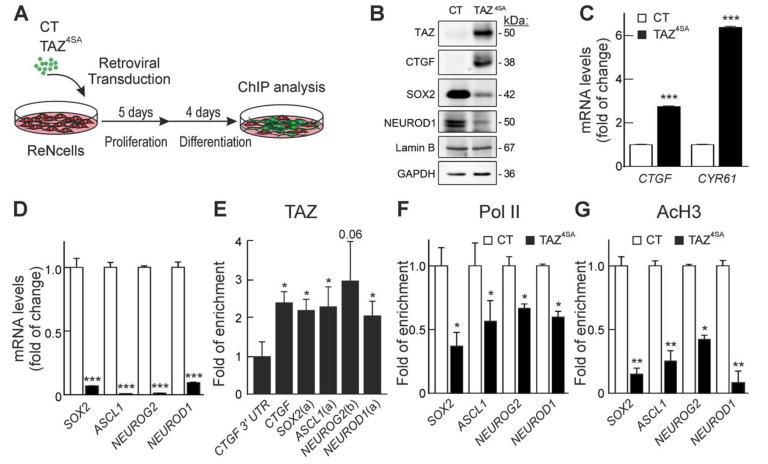
TAZ/TEAD induces epigenetic changes that inhibit the expression of SOX2 and proneuronal genes. (**A**) Representative scheme of experimental procedure. ReNcells VM were transduced with retroviral vector control (CT) or TAZ^4SA^ and grown for five days in proliferative conditions and 4 days in differentiation conditions; (**B**) representative immunoblots of TAZ, CTGF, SOX2, and NEUROD1 carrying GAPDH and Lamin B as loading controls; (**C**) mRNA levels of TAZ targets *CTGF* and *CYR61*; (**D**) mRNA levels of *SOX2*, *ASCL1*, *NEUROG2* and *NEUROD1*. mRNA levels were determined by qRT-PCR and normalized by the geometric mean of *ACTB*, *GAPDH,* and *TBP* levels. Data are mean ± S.D. (*n* = 4). Statistical analysis was performed using Student’s *t*-test. *** *p* < 0.001 vs. CT. (**E**) ChIP-qPCR analysis of putative TAZ/TEAD interacting regions in *SOX2* and proneurogenic the genes as indicated in Table A2 and Table A4 of the Appendix A. (**F**,**G**) ChIP-qPCR analysis of enrichment of Pol II (**F**) or AcH3 (**G**) in the genes *SOX2*, *ASCL1*, *NEUROG2,* and *NEUROD1*, immunoprecipitated with anti-Pol II or anti-AcH3 antibodies, respectively. qPCR determination was performed with the oligonucleotides indicated in Table A2 of the Appendix A. Data represent mean ± S.D. of three independent immunoprecipitations. Statistical analysis was performed using one-way ANOVA. * *p* < 0.05 vs. each control group.

In order to validate at least some of the TAZ/TEAD-interacting regions, we performed chromatin immunoprecipitation assays (ChIPs) in TAZ^4SA^ expressing ReNcells maintained for five days in proliferation medium and four additional days in differentiation medium (Figure 8A). These cells presented and increase in CTGF and a decrease in SOX2 and NEUROD1 protein levels (Figure 8B) as well as an increase in the bona fide TAZ-regulated *CTGF* and *CYR61* transcripts (Figure 8C) and a decrease in the proneurogenic transcripts (Figure 8D). For the ChIP-qPCR analyses, we used as control for normalization a fragment of the *CTGF* 3’ untranslated region (*CTGF* 3′UTR) that does not bind TAZ/TEAD. We found TAZ enrichment in the positive control *CTGF* binding region of TAZ/TEAD that has been characterized previously [49,50] but also in the TAZ/TEAD sequences of the proneuronal genes *SOX2*, *ASCL1*, *NEUROG2,* and *NEUROD1* (Figure 8E), described in Table A4 of the Appendix A.

### 3.6. TAZ induces Epigenetic Changes at the Regulatory Regions of Proneurogenic Genes

To further explore the repressor effect of TAZ on the expression of proneuronal genes, we investigated the recruitment of RNA polymerase II (Pol II) to the regulatory regions of these genes, because Pol II is engaged together with the transcription machinery [51,52]. A ChIP assay was performed by immunoprecipitating Pol II followed by qPCR with oligonucleotides corresponding to the TAZ/TEAD regions of *SOX2*, *ASCL1*, *NEUROG2,* and *NEUROD1* (Figure 8F). Pol II occupancy was significantly reduced at regulatory regions of the proneurogenic genes in TAZ^4SA^-expressing cells compared to control cells.

We then surveyed major histone modifications. Histone 3 (H3) acetylation at the *n*-terminal Lysine 9 is generally associated with gene activation [53,54]. ChIP-qPCR analysis of the TAZ/TEAD sites using anti-acetylated Lys9-H3 antibody (AcH3) indicated that H3 acetylation was reduced by TAZ^4SA^ overexpression in proneurogenic genes (Figure 8G).

## 4. Discussion

Genetic programs aimed at maintenance of stemness vs. commitment to differentiation into specific cellular lineages are tightly governed by epigenetic modifications and remodeling of chromatin. At this time, the specific factors that participate in the separation of both programs are still a matter of study [55,56]. Recent studies have suggested that the YAP and TAZ effectors for the WNT and Hippo pathways mediate epigenetic modifications in association with the chromatin-remodeling proteins, therefore affecting accessibility and activity of target genes [57]. Considering that the WNT and Hippo pathways participate in the maintenance of the NSPCs pool [58], in this study, we investigated the specific role of TAZ in neurogenesis.

Our immunofluorescence analysis in vivo and in vitro indicated a negative correlation between TAZ expression and the neurogenic commitment and differentiation of neuronal progenitors. Although no previous analyses were focused in TAZ expression in neurogenic niches, our findings are in line with the downregulation of the paralog YAP during neuronal differentiation [59,60].

The longitudinal study of the SVZ and SGZ dynamics was consistent with a common role of TAZ in both neurogenic niches, as its levels decreased in parallel to the exhaustion of NSPCs. However, we also observed differences in the proportion of differentiated nerve cell phenotypes. This could be attributed to the particular topology of each neurogenic niche and other non-NSPC autonomous local effects such as the influence of ependymal cells that displace NSPCs from the ventricular zone into the SVZ and further into striatum or cortex [61]. In contrast, the hippocampal NSPCs exit the cell cycle after several asymmetric divisions to produce a dividing progeny destined to become neurons and subsequently convert into mature astrocytes that migrate to the granular layer [62]. In order to concentrate our study on the common mechanisms of NSCPs dynamics, we used the midbrain-derived immortalized NSPC line ReNcell VM which shares general features of NSPCs. It is interesting that mechanistically these cells exhibited essentially a similar response to TAZ regulation as the NSPCs of the adult neurogenic niches.

We have performed a bioinformatics analysis to determine if TAZ/TEAD might bind the promoters of *SOX2*, *ASCL1*, *NEUROG2*, and *NEUROD1* and exert direct repression and found several putative TAZ/TEAD interaction regions. Further studies will be required to determine if all these sites are indeed responsible for direct negative regulation by TAZ/TEAD, but, with the exception of *NEUROG2* that did not reach statistical significance, our present study has validated at least one for each of them. Recent studies have also suggested a repressor function of TAZ in differentiation of cancer cells [63] and in the negative regulation of peroxisome proliferator-activated receptor-γ in mesenchymal stem cell differentiation [64], and NFAT5 in response to hyperosmotic stress and IL1β in inflammation [65]. Therefore, our study extends these findings to the commitment of NSPCs towards neuronal differentiation.

The relevance of repression of *SOX2* and proneuronal genes is suggested by the fact that these factors exhibit a rapid turnover, therefore implying the need for continuous gene transcription [66,67]. Interestingly, ASCL1 targets the transcription factors TEAD1, TEAD2, and *WWTR1* (TAZ) in the developing ventral mesencephalon [68], suggesting a mutual regulation by TAZ vs. at least this proneuronal factor. An additional layer of connectivity is the capacity of proneuronal factors to regulate their own expression. For example, SOX2 can enhance the expression of *ASCL1* and *NEUROG2* by cooperating with RMST (Rhabdomyosarcoma 2 Associated Transcript) [27] and POU3F2 (POU domain, class 3, transcription factor 2) [69]. On the other hand, NEUROG2 upregulates *SOX4*, which co-activates *NEUROD1* and *NEUROD4* [70].

The regulation of SOX2 expression by TAZ brings important consequences for neuronal differentiation vs. maintenance of neural stemness. SOX2 is required to differentiate neural crest cells into dorsal root ganglion neurons because specific ablation of SOX2 in the migratory neural crest reduced by half the number of neurons in the dorsal root ganglion of chicken and E14.5 mouse embryos [71]. In the same study, it was reported that SOX2 induces the expression of NEUROG1 and ASCL1 genes further demonstrating a role of SOX2 in the commitment of neural stem cells towards neuronal differentiation. Therefore, our observed downregulation of SOX2 by TAZ might further contribute to loss of expression of at least these proneuronal genes. On the other hand, SOX2 participates in cellular reprogramming of mouse and human fibroblasts into multipotent neural stem cells [72] and human pericytes can be reprogrammed into neuronal cells by retrovirus-mediated co-expression of SOX2 and ASCL1 [72]. Taken together, these observations suggest that SOX2 maintains neural progenitor identity [73] and might cooperate with lineage-defined factors to facilitate differentiation of subtype-specific neurons. This dual role of SOX2 is probably coordinated with a network of transcription factors and local signals that operate under different circumstances [74]. In fact, both knock-down of SOX2 and its overexpression block the self-renewal of neural stem cells and lead to their differentiation [74,75,76,77].

We have shown that TAZ depletion in NSPCs leads to a loss of stemness. This observation tightly correlates with the longitudinal study of TAZ expression shown in Figure 1 and Figure 2, indicating that the TAZ protein levels decline with aging, consistent with reports showing that negative regulators of TAZ increase with aging. For instance, GSK-3 is a protein kinase whose activity is increased in the elderly and in some neurodegenerative diseases such as Alzheimer’s disease [78]. It was found previously that GSK-3 phosphorylates TAZ, thus creating a recognition site for β-TrCP-mediated ubiquitination and proteasome degradation [79]. In another study, the transcription factor NRF2, master regulator of multiple cytoprotective responses, induced TAZ expression [80]. NRF2 transcriptional activity declines with aging and consistently TAZ levels decline as well. A consequence of TAZ downregulation is the progressive differentiation of the NSPCs and the exhaustion of the neurogenic niche. Then, in old mice, all NSPCs are expected to be differentiated and the neurogenic niches have disappeared. This observation implies that interventions aimed at maintaining TAZ expression longer during life would result in prolongation of neurogenic capacity for self-renewal. However, it must be considered that overexpression of TAZ is a hallmark of glioblastomas [80], which may limit the validity of this approach. Although in this study we explored the role of TAZ in repression of neuronal differentiation, it must be noted that this transcription co-factor may have additional roles in other nerve cells. According to the Brain RNA-seq database, TAZ is highly expressed in human and murine endothelial cells and astrocytes [81,82].

The participation of the WNT and Hippo pathways in the homeostasis of the neurogenic niches is deeply influenced by inflammatory and oxidative stress signals. However, the impact of these signals on TAZ is not defined yet. In a previous study, we showed that the transcription factor NRF2, a key regulator of antioxidant and anti-inflammatory responses activates TAZ in glioblastomas stem cells [80], suggesting that TAZ might participate in stem cell fate in response to oxidative stress. Similarly, the Ser/Thr protein kinase GSK-3, instrumental in the canonical WNT pathway, is activated by inflammatory and oxidative stress signals [83] and leads to phosphorylation and degradation of TAZ [79]. Our study shows that TAZ levels decrease with aging in the neurogenic niches, and provides a basis for a future study on its participation in inflammatory and oxidative stress signals, which are present in the neurogenic microenvironment and influence NSPCs’ auto renovation and differentiation in age-related neurodegenerative diseases.

Contrary to the effect on *bona fide* TAZ target genes, such as *CTGF* and *CYR61*, involved in proliferation, here TAZ represses genes involved in neuronal differentiation. Under proliferative conditions’ TEADs, in complex with YAP or TAZ, induce gene transcription via proximal promoters and distal enhancers that are marked by histone H3 acetylation [50]. In addition, under proliferative conditions, TEAD-YAP complexes recruit the Mediator complex to specific gene enhancers, allowing the recruitment of the CDK9 elongating kinase [84]. However, recent studies reported that the Drosophila ortholog Yki is found in components of the chromatin-remodeling and histone methyltransferase complexes [85,86,87,88]. Kim et al. described the interaction between the other Hippo effector, YAP, and nucleosome remodeling and histone deacetylase (NuRD) complex [89]. More relevant to our observations, TAZ/TEAD directly interacts with the histone deacetylation complex for suppression of ΔNp63 transcription [63], in skin development and adult stem/progenitor cell regulation. Together, these studies support our observations and suggest that, under non-proliferative conditions, TAZ may participate in the gene repression. Our study potentially opens a new path for understanding the role of TAZ in NSPCs fate and neuronal programming.

## Appendix A

**Table A1:** Primary antibodies.

Antibody	Reference	Clonality	Isotype	Dilution and Application
**Acetyl-Histone H3**	Sigma Aldrich. 06-599.	Polyclonal	Rabbit	1.5 µg (ChIP)
**CTGF (L-20)**	Santa Cruz Biotechnology. Sc-14939.	Polyclonal	Goat	1/2000 (WB)
**Doublecortin**	Santa Cruz Biotechnology. Sc-8066.	Polyclonal	Goat	1/250 (IHC-Fr; ICC/IF)
**GAPDH**	Millipore. CB1001.	Monoclonal	Mouse	1/15000 (WB)
**GFAP**	Dako. Z0334.	Polyclonal	Rabbit	1/200 (IHC-Fr; ICC/IF)
**GFAP**	Sigma-Aldrich. G3893.	Monoclonal	Mouse	1/200 (IHC-Fr; ICC/IF)
**IgG2a**	Abcam. Ab18413.	Monoclonal	Mouse	1/250 (ChIP)
**Lamin B**	Santa Cruz Biotechnology. Sc-6217.	Polyclonal	Goat	1/2000 (WB)
**MAP2**	Sigma-Aldrich. M9942.	Monoclonal	Mouse	1/200 (ICC/IF)
**Nestin**	Abcam. Ab11306.	Monoclonal	Mouse	1/200 (IHC-Fr; ICC/IF)
**Nestin**	Novus Biologicals. NB100-1604.	Polyclonal	Chicken	1/500 (IHC-Fr; ICC/IF)
**NEUROD (A-10)**	Santa Cruz Biotechnology. Sc-46684.	Monoclonal	Mouse	1/500 (WB)
**RNA Pol II (A-10)**	Santa Cruz Biotechnology. Sc-17798.	Monoclonal	Mouse	3 µg (ChIP)
**Rabbit IgG**	Abcam. Ab37415.	Polyclonal	Rabbit	1/100 (ChIP)
**SOX2**	R&D Systems. AF2018.	Polyclonal	Goat	1/500 (IHC-Fr; ICC/IF) 1/2000 (WB)
**TAZ**	Sigma Aldrich. HPA007415.	Polyclonal	Rabbit	1/2000 (WB) 1/200 (IHC-Fr; ICC/IF) 1/100 (ChIP)
**TUBB3**	Sigma-Aldrich. T2200.	Polyclonal	Rabbit	1/200 (ICC/IF)
**V5**	Life Technologies. 37-7500.	Monoclonal	Mouse	1/2000 (WB)
**YAP/TAZ (D24E4)**	Cell Signaling Technology. 8418.	Monoclonal	Rabbit	1/2000 (WB)

**Table A2:** Oligonucleotides used for ChIP-qPCR of RNA Pol II and acetylated H3. For each gene, ChIP-qPCRs were per formed with the indicated amplimers which contain putative TEAD sequences. The letters in brackets correspond to the TEAD sequences depicted in Table A4 of the Appendix A.

GENE PRODUCT	FORWARD PRIMER (5′- 3′)	REVERSE PRIMER (5′- 3′)	bp
***SOX2* (a)**	CATTTGAAAGCCGCACGACC	ATGCTTCTACTGTCTGCCCC	83
***ASCL1* (a)**	TTTGGGTGCTCACCTCCTAT	GGATTCACACCTCAGGCCTTT	87
***NEUROG2* (b)**	TGTTTTGTTAGAGGGGCAGGT	GCCTAAATTTCCACGCTTGCAT	83
***NEUROD1* (a)**	CTAACTGGCGACAGATGGGC	CATTTGTATGCCGCGGAGC	75

**Table A3:** Oligonucleotides used for qRT-PCR.

GENE PRODUCT	FORWARD PRIMER (5′- 3′)	REVERSE PRIMER (5′- 3′)
***ACTB***	TCCTTCCTGGGCATGGAG	AGGAGGAGCAATGATCTTGATCTT
***ASCL1***	CATCTCCCCCAACTACTCCA	GAAAGCCATGTCTCTCAGGC
***CTGF***	CTTCTGTGACTTCGGCTCCC	GATGCAGGGAGCACCATCTT
***CYR61***	CCAAGAAATCCCCCGAACCA	CGGAACCGCATCTTCACAGT
***GAPDH***	CTCTCTGCTCCTCCTGTTCGAC	TGAGCGATGTGGCTCGGCT
***NEUROD1***	GGTGCCTTGCTATTCTAAGACGC	GCAAAGCGTCTGAACGAAGGAG
***NEUROG2***	ATCCGAGCAGCACTAACACG	GCTGAGGCACAGTTAGAGCC
***SOX2***	GAGCTTTGCAGGAAGTTTGC	GCAAGAAGCCTCTCCTTGAA
***TBP***	TGCACAGGAGCCAAGAGTGAA	CACATCACAGCTCCCCACCA
***TEAD1***	CTGAGTCGCAGTTACCACCA	AGCCTGGAGCCTTTTCAAG
***TEAD2***	ACATGATGAACAGCGTCCTG	CAGCAGTTCCTGGGTGTCTC
***TEAD3***	CATCGAGCAGAGCTTCCAG	CGTGCAATCAACTCATTTCG
***TEAD4***	GCCTTCCACAGTAGCATGG	AAAGCTCCTTGCCAAAACC
***WWTR1***	TTTCCTCAATGGAGGGCCA	GGGTGTTTGTCCTGCGTTTT

**Table A4:** Putative TEAD binding sites in the promoter regions of the proneurogenic genes. A PSSM was generated from the JASPAR consensus sequence for binding of the four TEAD paralogs, and used to scan these genes according to a Phython 3.4-based bioinformatics program (available upon request). Putative sequences were then curated with the following criteria: (1) from the ENCODE data base, putative sites were selected on the basis of their location in DNAse I and H3K27Ac sensitive regions, indicative of being regulatory regions; (2) only one mismatch was allowed in the core consensus sequence CATTCC (underlined). The table shows chromosome location, putative TEAD2-binding sequence, maximal score (MS) and relative score (RS) (>80%) of each TEAD isoform according to the Python program. Blanc cells indicate that the program did not recognize a certain sequence for a certain TEAD isoform with a threshold >80%. The letters in brackets in the “Sequence” column correspond to the core of the fragments that were found by ChIP-pPCR with anti-TAZ antibody (See Table A2 of the Appendix A for ChIP primers and PCR fragment length). TEAD2 shows the most robust data and, as shown in Figure A2 of the Appendix A, this is the isoform preferentially expressed in ReNcells VM.

GENE	LOCALIZATION	SEQUENCE (FROM TEAD2)	TEAD2		TEAD1		TEAD3		TEAD4	
			MS	RS	MS	RS	MS	RS	MS	RS
***SOX2***	Chr3: 181428844-181428831	CCCCATTCCCATC (a)	8.49	90.9	-		-		-	
	chr3:181431413-181431401	TCCATTCCCCCG (b)	6.99	89.3	13.88	83	2.17	92.9	-	
	chr3:181428382-181428394	AAGATTCCTGAG (c)	8.06	90.5	13.77	82.8	8.01	95.8	8.78	90.8
***ASCL1***	chr12:103349820-103349833	CCACATACCAAGA (a)	9.6	92	-		12.2	97.8	12.62	94.6
	chr12:103349554-103349542	CACATCCCTGAC (b)	9.57	92	13.99	83.1	-		-	
	chr12:103354060-103354047	CAACATTTCTATA (c)	7.77	90.1	-		-		-	
***NEUROG2***	chr4:113435415-113435427	TTGCATTCCCCCT (a)	9.07	91.5	14.3	83.6	9.23	96.4	10.91	92.9
	chr4:113435345-113435358	ATACATTTCTTTC (a)	9.31	91.8	-		-		-	
	chr4:113435031-113435044	TTGCATTCAATCA (b)	5.56	87.9	-		-		-	
***NEUROD1***	chr2:182545564-182545577	CTCCATTCCGGCC (a)	9.07	91.5	-		-		-	
	chr2:182543940-182543952	TACATTTCAGCG (b)	10.29	92.8	15.41	85.4	-		8.8	90.8
	chr2:182544023-182544035	CACATTCTACTT (c)	9.16	91.6	15.78	86	-		13.72	95.6

## Appendix B

Script created with Python to scan in human genes the putative TEADs-binding sequences with the PSSM and calculate their relative score. Green letters indicate the variable that needs to be changed for each transcription factor: |Max| and |Min| correspond to absolute value of maximum and minimum calculated from PSSM matrix. N corresponds to the number of positions of PSSM matrix. PSSM is obtained from the frequency matrix of each transcription factor from the JASPAR database.

import sys

import re

**def** Get_PSSM(FileName):

 tmp={}

 MyFile=open(FileName,‘r’)

 **for** Nt **in** [“A”,“C”,“G”,“T”]:

  pos=MyFile.readline()

  tmp[Nt]=pos.strip().split(“\t”)

 MyFile.close()

 **return**(tmp)

**def** Get_Coordin(Coord):

 MyRE=r’(\w*)(chr[0-9XY]{1,2}):(\d+)-(\d+)’

 MyReg=re.compile(MyRE)

 Res=MyReg.search(Coord)

 **return**(Res.groups())

**def** Get_MaxScore(Seq,PSSM):

 MaxSc=-100000

 book={}

 **for** idx **in**range(len(Seq)-len(PSSM[“A”])+1):

  tmpSc=0

  sseq=“”

  sequence=[]

  sequence2=[]

  **for** pos **in**range(len(PSSM[“A”])):

   tmpSc=tmpSc+float(PSSM[Seq[idx+pos]][pos])

   sseq=sseq + Seq[idx+pos]

  **if** MaxSc<tmpSc:

   MaxSc=tmpSc

   sequence.append(str(tmpSc))

   sequence2.append(sseq)

   book=dict(list(book.items()) + list((dict(zip(sequence,sequence2))).items()))

 **return**(MaxSc)

**def** Get_Seq(Seq,PSSM):

 MaxSc=-100000

 book={}

 **for** idx in range(len(Seq)-len(PSSM[“A”])+1):

  tmpSc=0

  sseq=“”

  sequence=[]

  sequence2=[]

  **for** pos **in**range(len(PSSM[“A”])):

   tmpSc=tmpSc+float(PSSM[Seq[idx+pos]][pos])

   sseq=sseq + Seq[idx+pos]

  **if** MaxSc<tmpSc:

   MaxSc=tmpSc

   sequence.append(str(tmpSc))

   sequence2.append(sseq)

   book=dict(list(book.items()) + list((dict(zip(sequence,sequence2))).items()))

 **return**(book[str(MaxSc)]))

**def** Main(Args):

 PSSM={}

 PSSM=Get_PSSM(Args[1])

 MyFile=open(Args[2],‘r’)

 **for** Line **in** MyFile:

  Fields=Line.strip().split(“\t”)

  (Name,Chr,Str,End)=Get_Coordin(Fields[0])

  directseq=Fields[1]

  basecomplement={“A”:“T”:“C”:“G”,“T”:“A”:“G”:“C”}

  rDNAsense=directseq[::-1]

  antisenseDNA=“”

  **for** base **in** rDNAsense:

   antisenseDNA = antisenseDNA+basecomplement[base]

  MaxScore=Get_MaxScore(Fields[1].upper(),PSSM)

  MaxScore2=Get_MaxScore(antisenseDNA.upper(),PSSM)

  Sequence=Get_Seq(Fields[1].upper(),PSSM)

  Sequence2=Get_Seq(antisenseDNA.upper(),PSSM)

  finded= Fields[1].find(Sequence)

  finded2= antisenseDNA.find(Sequence2)

  **if**int(Str)>int(End):

   **print**(Name,“\t”,Chr,“\t”,str(int(Str)-finded),“\t”,str(int(Str)-finded-**N**),“\t”,

    round(MaxScore,2),“\t”,round(MaxScore+**|Min|**)/(**|Max|**+**|Min|**),3),“\t”,

    Sequence,“\t”, str(finded2+int(End)),“\t”, str(finded2+int(End)+**N**),“\t”, round(MaxScore2,2),“\t”,round(MaxScore2+**|Min|**)/(**|Max|**+**|Min|**),3),“\t”,Sequence2)

  **else:**

   **print**(Name,“\t”,Chr,“\t”,str(finded+int(Str)),“\t”,str(finded+int(Str)+**N**),“\t”,

    round(MaxScore,2),“\t”,round(MaxScore+**|Min|**)/(**|Max|**+**|Min|**),3),“\t”,

    Sequence,“\t”,str(int(End)-finded2),“\t”,str(int(End)-finded2-**N**),“\t”,

    round(MaxScore2,2),“\t”,round(MaxScore2+**|Min|**)/(**|Max|**+**|Min|**),3),“\t”,Sequence2)

 MyFile.close()

**if**__name__==‘__main__’:

 **if**len(sys.argv)<2:

  **print**(“You must provide the name of the files containing the sequences and PSSM”)

 **else:**

  Main(sys.argv)

sys.exit()
